# Integrative Transcriptomic, Network, and Genomic Analysis of Peripheral Blood Mononuclear Cells Identifies Candidate Genes Associated with Dupilumab Clinical Response in Atopic Dermatitis Patients

**DOI:** 10.3390/ijms27115147

**Published:** 2026-06-05

**Authors:** Martina Krušič, Mario Gorenjak, Uroš Potočnik, Maruška Marovt

**Affiliations:** 1 Center for Human Molecular Genetics and Pharmacogenomics, Faculty of Medicine, University of Maribor, Taborska ulica 8, 2000 Maribor, Slovenia; 2 Faculty of Chemistry and Chemical Engineering, University of Maribor, Smetanova ulica 17, 2000 Maribor, Slovenia; 3 Department for Science and Research, University Clinical Centre Maribor, Ljubljanska ulica 5, 2000 Maribor, Slovenia; 4 Faculty of Medicine, University of Maribor, Taborska ulica 8, 2000 Maribor, Slovenia; 5 Department of Dermatovenerology, University Clinical Centre Maribor, Ljubljanska ulica 5, 2000 Maribor, Slovenia

**Keywords:** atopic dermatitis, transcriptomics, blood biomarkers, dupilumab

## Abstract

Atopic dermatitis (AD) is among the most common chronic inflammatory diseases. Due to the heterogeneous presentation of AD, patient response to treatment may differ considerably. Therefore, there is a pressing need for biomarkers associated with response to biological therapies. Thus, we aimed to identify blood-based candidate biomarkers associated with response in patients treated with dupilumab. The present study applied a multi-stage integrative analytical framework combining transcriptomic profiling, functional enrichment, co-expression network analysis, and genomic variant analysis to identify potential biomarkers. Eighteen dupilumab-naïve patients were enrolled in the transcriptomic analysis, with blood samples collected at baseline and after 16–18 weeks of therapy; five patients were identified as non-responders. Additionally, genotyping was performed in 34 patients. We identified a set of candidate genes (*RPL18A*, *RPS28*, *FAU*, *MASTL*, *AURKA*, *TAF2*, *BUB1B*, and *RNF135*) and genomic variants that may reflect underlying biological mechanisms influencing therapeutic response. However, given the limited sample size, these findings should be considered exploratory and hypothesis-generating. Finally, our study identified exploratory candidate genes potentially associated with variability in dupilumab treatment response. Moreover, our study represents an incremental contribution to existing knowledge, opening avenues for research that may ultimately lead to personalized medicine.

## 1. Introduction

Atopic dermatitis (AD) is the most common chronic inflammatory skin disease [1,2,3], affecting approximately 20% of children and 10% of adults worldwide [4]. Research findings indicate an increasing incidence and prevalence of the disease [5], with environmental factors also contributing significantly [6].

The disease is heterogeneous, which is reflected in a wide spectrum of clinical characteristics. Among the most common symptoms are eczematous lesions, severe itching, and dry skin [2,7]. Pruritus, one of the most characteristic symptoms, drives patients to scratch, which consequently leads to sleep disturbances and further exacerbation of the disease, thereby significantly affecting patients’ quality of life [8,9].

Due to the heterogeneous nature of AD, it is expected that patients will respond differently to treatment. A set of evaluated genetic biomarkers represents a pivotal tool for the development of personalized medicine [10,11]. In recent years, the number of biological therapies has increased substantially, leading to a growing need to identify reliable biological markers of treatment response.

Despite effective therapies, more than 55% of adult patients with moderate to severe AD report inadequate disease control [12,13]. Although many studies are not yet applicable in clinical practice, each contributes new insights and a deeper understanding of the pathogenesis of AD and opens opportunities for the identification of new therapeutic targets.

Through a comprehensive literature review, we observed a notable lack of studies investigating the blood transcriptome in patients with AD treated with dupilumab. In particular, there is a lack of studies that examine differences in blood transcriptome profiles between responders and non-responders to dupilumab treatment. In line with this, our study aimed to identify potential biomarkers of treatment response in the blood of patients with moderate-to-severe AD. By focusing on peripheral blood, an easily accessible and minimally invasive source, we sought to characterize gene expression differences between responders and non-responders and thereby contribute to the development of response-associated molecular tools for personalized treatment strategies. Because of the exploratory design and limited cohort size, the present study was intended primarily for candidate biomarker discovery and biological hypothesis generation. Importantly, all patients included in the transcriptomic analysis were naïve with respect to dupilumab therapy, allowing the identification of baseline molecular signatures that are not confounded by prior biologic treatment effects.

## 2. Results

### 2.1. RNA Sequencing Analysis

To detect gene expression signals potentially masked by treatment and ongoing inflammation, we conducted a three-stage RNA-seq analysis (Supplementary Figure S1).

First, we performed stage one differential gene expression analysis. In this stage, we compared responders’ follow-up samples with healthy controls to identify differentially expressed genes influenced by dupilumab therapy. This analysis revealed 4,526 differentially expressed genes with an adjusted *p*-value < 0.05. ENTREZ IDs of genes identified in stage one are presented in Supplementary Table S1.

We removed significant therapy-related genes identified in stage one before proceeding to stage two. Stage two included two substages aimed at identifying differentially expressed genes associated with inflammation. In the first substage, we compared non-responders with responders’ follow-up samples, whereas in the second substage, we compared non-responders’ follow-up samples with healthy controls. We identified 383 differentially expressed genes in substage one and 572 genes in substage two, all with *p*-value < 0.05. We combined the unique differentially expressed genes identified in both substages to define inflammation-related genes and removed them from the analysis before proceeding to stage three. This set included 929 unique genes (ENTREZ IDs are presented in Supplementary Table S2).

Prior to stage three, we removed previously identified inflammation-related genes. Thus, stage three was performed on the full gene set, excluding only the inflammation-associated genes identified in stage two, as the samples were obtained prior to therapy (naïve to dupilumab treatment). In stage three, we compared non-responders’ and responders’ baseline samples and revealed 817 significantly differentially expressed genes (*p*-value < 0.05). The identified genes represent a first step toward identifying candidate molecular signals associated with dupilumab response. Stage three differentially expressed genes are presented in Supplementary Table S3.

### 2.2. Gene Ontology Analysis

In addition, we carried out gene ontology analysis (GO) on statistically significant differentially expressed response-associated genes. Of the 817 genes, we excluded from the analysis those genes that did not have a symbol. In total, we included 791 statistically significant genes in the GO analyses, 338 upregulated and 453 downregulated. Furthermore, we performed GO analysis for molecular function and biological processes separately for up- and downregulated genes. In the enrichment analysis, the upregulated genes were associated with one GO term related to molecular function, which comprised 12 unique genes. No GO terms related to biological processes were identified for the upregulated genes. In contrast, the downregulated genes were associated with 56 GO terms related to molecular function, comprising 108 unique genes. Additionally, 56 GO terms related to biological processes were identified for the downregulated genes, including 133 unique genes. In total, we obtained 181 unique genes listed at the enriched GO terms. All identified GO terms are presented in the Supplementary Table S4.

Genes listed in the GO analysis were excluded from the set of differentially statistically significant genes to obtain the subset of genes not represented in any enriched GO term (non-GO-enriched genes). This resulted in 326 upregulated and 272 downregulated genes (Supplementary Tables S5 and S6).

### 2.3. Gene Scores and Association Testing

After GO analysis, we calculated gene score variables for each individual for both GO terms and non-GO-enriched gene terms to enable association testing with the outcome. Thereafter, we used bias-reduced logistic regression, where we fitted a model with dupilumab response as the dependent outcome variable and gene score adjusted for sex and age as independent variables. We performed regression models for each gene score variable separately.

For GO terms with upregulated genes, we identified one significant GO term (GO:0003735, *p*-value = 0.046) associated with the outcome. Moreover, gene scores derived from upregulated non-GO-enriched genes were also significantly associated with the outcome (*p*-value = 0.040). On the other hand, for GO terms with downregulated genes, we identified seven significant GO terms associated with the outcome (GO:0044839, *p*-value = 0.047; GO:0000086, *p*-value = 0.044; GO:1902749, *p*-value = 0.045; GO:0010389, *p*-value = 0.048; GO:1901990, *p*-value = 0.048; GO:0007093, *p*-value = 0.038; GO:1901991, *p*-value = 0.049), while no significant association was observed for non-GO-enriched genes.

Additionally, genes were extracted from GO terms whose gene scores were significantly associated with the outcome (*p*-value < 0.05). Finally, we obtained 36 unique genes from the enriched terms associated with the outcome (*RPL23*, *MRPL11*, *RPL18A*, *RPL22L1*, *RPS12*, *RPS28*, *RPL27*, *FAU*, *MRPL28*, *MRPS12*, *RPS23*, *RPL21*, *BLM*, *FBXO5*, *MASTL*, *AURKA*, *CCNA2*, *AXIP1*, *MRE11*, *CDC25A*, *NDC80*, *TAF2*, *CDK1*, *LATS1*, *KIF14*, *BARD1*, *TAOK1*, *CDC14A*, *RBL1*, *ZW10*, *ECD*, *ANKRD17*, *BUB1B*, *CDC73*, *GIGYF2*, and *ATF2*).

### 2.4. Weighted Gene Co-Expression Network Analysis

For non-GO-enriched genes, we performed weighted gene co-expression network analysis (WGCNA) on 326 significantly upregulated genes derived from the non-enriched gene set, showing a significant gene score association with the outcome. We applied the WGCNA algorithm to construct gene co-expression networks and selected a soft-thresholding power corresponding to a scale-free topology fit of R^2^ = 0.8. Next, we identified gene modules through average hierarchical clustering combined with dynamic tree cutting. We set the minimum module size to 20. In total, we obtained five distinct modules, comprising 14 (gray), 109 (turquoise), 104 (blue), 53 (brown), and 46 (yellow) genes (Supplementary Figure S2). The gray module 0 contained genes that were not assigned to any co-expression module.

Furthermore, we screened the modules for the promising hub genes. Therefore, we calculated gene expression profiles for each given module. For each module, we performed bias-reduced logistic regression to assess the association with the outcome, using the module gene score as the predictor and adjusting for sex and age. Regression models were fitted separately for each module, with the blue module 2 showing the strongest tendency of association with the outcome (*p*-value = 0.0519). All observed modules, the number of genes in each module, and *p*-values for outcome association of each module are listed in Table 1.

For module 2, module membership (kME) was calculated for each gene to assess its correlation with the module eigengene. Genes with kME > 0.9 were considered highly connected hub genes and were selected for further analyses. Based on this, we identified four hub genes, *SLC2A6*, *HMOX1*, *RNF135*, and *ADAP1*.

### 2.5. Genomic Profiling and Association Analysis

A total of 40 genes were identified and subsequently used as targets for genomic integration analysis. We performed genomic profiling on eighteen naïve patients and an additional sixteen long-term responders. First, a genome-wide association analysis was conducted in response to dupilumab therapy. Integration was performed by extracting variants located within ±100 kb of the 40 target genes. Statistically significant (*p*-value < 0.05) targeted genomic signals were found in the gene regions of *RPL18A*, *RPS28*, *FAU*, *MASTL*, *AURKA*, *TAF2*, *BUB1B*, *ATF2*, and *RNF135*. All identified variants were pruned at an R^2^ threshold of 0.5 to retain independent signals, resulting in a final set of 10 variants across 9 genes (Table 2). For all SNPs, regional Manhattan plots were created and are presented in the Supplementary Figures S3–S11.

Additionally, we retrieved eQTL information from publicly available data sources, the GTEx Portal and the eQTL Catalogue, to confirm gene–SNP interactions. One identified variant, rs590142, showed a significant eQTL association with gene expression in whole blood. For two SNPs in the *ATF2* gene (rs2788522 and rs12613111), no eQTL data or proxy SNPs expressed in blood or blood cells were identified. Therefore, these variants were excluded from downstream analyses. For an additional seven SNPs, proxy variants (D’ > 0.7) showing eQTL associations with gene expression in blood or blood cells were identified. eQTL data obtained for selected variants is present in Table 3.

Based on the retained eQTL-supported variants, Mendelian randomization (MR) analysis using the inverse-variance weighted method was subsequently performed to further explore the association of genes and the outcome through variants as instrumental variables.

MR analysis suggested potential associations between genetically predicted gene expression and dupilumab treatment response for all analyzed genes (*RPL18A*, *RPS28*, *FAU*, *MASTL*, *AURKA*, *TAF2*, *BUB1B*, and *RNF135*), with all associations reaching statistical significance (*p*-value < 0.05). As only one blood-related eQTL instrument was available for each gene, MR was performed using single-SNP instruments and therefore provided only limited support for causality.

### 2.6. Exploratory External Dataset Comparison

To further explore the consistency of the identified transcriptional signals, we analyzed an independent dataset from the Gene Expression Omnibus (GEO), GSE208405, comprising 46 samples (six non-responders and 40 responders). Several investigated genes showed nominal expression differences between responder groups, with *RPS28* demonstrating the strongest signal among the analyzed candidate genes (Table 4). Because clinically defined dupilumab response groups were not available, responder status was inferred indirectly from eosinophil-associated transcriptomic patterns. Therefore, this analysis should be interpreted as an exploratory external comparison rather than a formal validation.

### 2.7. RT-qPCR Assessment of Candidate Genes

**Figure 1:**
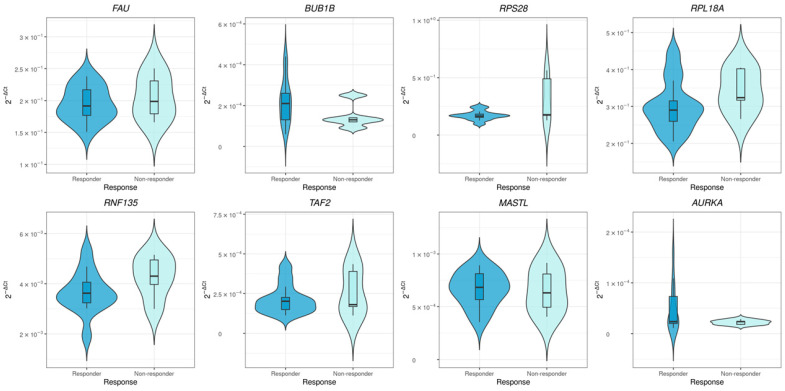
Violin plots of 2^−ΔCt^ expression for selected genes.

To further assess expression patterns of the prioritized candidate genes, reverse transcription quantitative polymerase chain reaction (RT-qPCR) was performed. Relative gene expression (2^−ΔΔCt^) was calculated between the non-responders relative to the responders and is presented in Table 5 (Figure 1). Logistic regression was performed. None of the investigated genes reached statistical significance after logistic regression (Table 5). However, *BUB1B* showed suggestive significance (*p*-value = 0.099) in the logistic regression model. Importantly, *BUB1B*, *AURKA*, and *RPS28* showed concordant expression direction compared with the RNA-seq analysis.

## 3. Discussion

To the best of our knowledge, few studies have applied a multi-stage integrative approach combining RNA-seq, GO analysis, co-expression network modeling, and genomic profiling to investigate potential biomarkers of dupilumab response in AD. By systematically removing treatment- and inflammation-related genes, we aimed to uncover underlying gene expression patterns potentially associated with therapeutic response. For this reason, we used blood samples, as they are more accessible and less invasive compared to tissue biopsies.

The strength of the present study is the three-stage analytical approach, which was designed to reduce therapy- and inflammation-related signals that could obscure baseline response-associated molecular patterns. To sum up, first, we identified genes influenced by therapy by comparing responders’ follow-up samples with healthy controls. Subsequently, we removed therapy-related genes to unmask inflammation-associated genes, enabling a more defined analysis of baseline sample differences between responders and non-responders. Thus, an important strength of our study is that all patients included in the transcriptomic analysis were naïve with respect to dupilumab treatment, ensuring that the identified gene expression patterns reflect intrinsic baseline biology associated with treatment response rather than transcriptional changes induced by prior therapy or inflammation. Together, we identified 817 significant differentially expressed genes representing candidate transcriptional signals potentially associated with therapeutic response.

Furthermore, to enrich identified genes, we performed a detailed analysis of the molecular functions and biological processes associated with these genes. GO analysis of the upregulated genes identified a single significantly enriched GO term, “structural constituent of ribosome.” In contrast, GO analysis of molecular functions for downregulated genes revealed 56 terms predominantly associated with protein kinase activity, chromatin organization and epigenetic regulation, and DNA-related ATP-dependent processes, with additional enrichment in ubiquitin-mediated protein modification and cytoskeletal functions. Moreover, GO analysis of downregulated genes revealed 56 terms associated with biological processes, mainly involving cell cycle, DNA replication, and chromatin regulation.

Moreover, we calculated gene scores for each GO term and the set of non-GO-enriched genes. Thus, allowing us to test the association of each enriched GO term or non-GO-enriched set with dupilumab response as the outcome. After fitting the regression models, we obtained 36 genes listed at GO terms and non-GO-enriched genes associated with the outcome. For non-GO-enriched genes, we performed WGCNA to further identify the association drivers. We obtained five modules, of which one module showed the tendency for significance threshold (module 2 blue, *p*-value = 0.0519). This module consists of 104 genes. Finally, we identified four hub genes based on module kME (*SLC2A6*, *HMOX1*, *RNF135*, and *ADAP1*).

In addition, to integrate the aforementioned findings with genomic regions, we extracted variants located within ±100 kb of the total 40 identified genes. Downstream genomic integration yielded a final set of eight prioritized candidate genes: *RPL18A*, *RPS28*, *FAU*, *MASTL*, *AURKA*, *TAF2*, *BUB1B*, and *RNF135*.

To the best of our knowledge, none of these SNPs has been previously associated with AD. Among identified genes, *RPL18A*, *RPS28*, and *FAU* are listed in the enriched GO term “structural constituent of ribosome”. Those genes predominantly encode ribosomal proteins involved in protein synthesis and cellular metabolism. The identification of SNPs in these genes in our AD cohort points to a potential involvement of translational machinery in disease pathogenesis. This is consistent with emerging evidence that dysregulation of ribosomal function may contribute to altered immune responses and inflammatory processes [14]. *RPL18A* (Ribosomal protein L18a) was previously identified as a gene capable of promoting B-cell transformation [15]. Furthermore, B cells have been implicated in the pathogenesis of atopic dermatitis, particularly through regulatory B cells (Bregs), which can modulate inflammatory responses via IL-10–mediated suppression of eosinophil activation and skin inflammation [16,17]. In addition, it was shown that dupilumab treatment increases transitional B cells in severe asthma [18]. Thus, the identification of *RPL18A* in our study may indicate that B-cell-related pathways contribute to variability in dupilumab treatment response.

*RPS28* (Ribosomal protein S28) has also been recently identified as a hub gene in allergic rhinitis, an IgE-mediated inflammatory disease that shares immunological mechanisms with atopic dermatitis, suggesting that *RPS28* may contribute to pathways involved in allergic inflammation [19].

*FAU* (FAU ubiquitin-like and ribosomal protein S30 fusion) encodes a fusion protein composed of the ubiquitin-like protein FUBI and ribosomal protein S30, a component of the 40S ribosomal subunit involved in protein synthesis (NCBI Gene: https://www.ncbi.nlm.nih.gov/gene/2197, accessed on 24 March 2026). To our knowledge, beyond this mechanistic role in ribosomal function, FAU has not previously been implicated in immune-mediated diseases, and our findings represent the first evidence linking this gene to dupilumab treatment response in AD.

*TAF2* (TATA-box binding protein associated factor 2) encodes a subunit of the TFIID transcription initiation complex involved in RNA polymerase II–dependent gene transcription. Beyond this fundamental role in transcriptional regulation, its involvement in immune-mediated diseases has only been reported in the context of prognosis and treatment response in acute myeloid leukemia [20].

Most notably, the genes *AURKA* (Aurora kinase A) and *BUB1B* (BUB1 mitotic checkpoint serine/threonine kinase B), which were previously associated with AD [21], were also identified in the present study. *AURKA* belongs to the serine/threonine kinase family and is widely recognized in cancer research [22,23,24]. Moreover, research has shown that expression levels of *AURKA* are increased in psoriatic lesions compared to healthy controls [25]. Upregulation of *AURKA* in inflammatory skin conditions has been associated with enhanced activation of the AIM2 inflammasome and the AKT/mTOR pathway, leading to increased keratinocyte-mediated inflammation [25]. A recent study by Liu and colleagues [21], aimed to uncover molecular mechanisms and potential therapeutic targets in AD using bioinformatics and experimental validation. Their protein–protein interaction network analysis identified five hub genes (*AURKA*, *BUB1B*, *CCNB1*, *MELK*, and *TTK*) associated with AD progression. The analysis of immune cell infiltration revealed strong associations between these genes and several immune cell types, including activated CD4 T cells, Th2 cells, regulatory T cells (Tregs), and central memory CD8 T cells. They also showed that mRNA expression levels of *AURKA* and *BUB1B* are upregulated in AD lesions. Moreover, protein BUB1B promotes homologous recombination-mediated DNA damage repair in breast cancer cells [26]. It shows that BUB1B enhances DNA repair capacity by activating the PI3K/AKT signaling pathway, which supports cancer cell survival. The PI3K/AKT pathway plays a very important role in several inflammatory diseases, including AD [27,28]. Its role is well established in psoriasis [29]. Interestingly, it has been shown that treatment with baricitinib inhibits the activation of PI3K/AKT and mTOR signaling pathways [30]. Several studies have shown that targeting the PI3K/AKT/mTOR signaling pathway alleviates AD symptoms [31,32].

In addition, *MASTL* (Microtubule-associated serine/threonine kinase-like) has been shown to regulate the PI3K/AKT/mTOR signaling axis through the MASTL–PP2A/B55 kinase–phosphatase module, thereby modulating AKT activity and downstream metabolic and signaling processes [33]. Importantly, this kinase–phosphatase module links cell-cycle regulation with PI3K/AKT/mTOR signaling and contributes to feedback mechanisms that restrain AKT activity during sustained mTOR signaling [33]. Given the central role of the PI3K/AKT/mTOR pathway in inflammatory diseases, these observations further support the potential involvement of MASTL in pathways relevant to AD pathogenesis. Several genes identified in our study have been associated with the PI3K/AKT/mTOR signaling pathway, which has previously been proposed as a potential therapeutic target in AD [34]. Taken together, these observations support the involvement of *AURKA*, *BUB1B*, and *MASTL* in AD-related signaling pathways and highlight the relevance of our findings, identifying candidate genes potentially associated with dupilumab treatment response.

*RNF135* was the only gene identified within the non-GO-enriched gene set. This is particularly interesting because it was not associated with any enriched GO terms, indicating that its potential involvement in AD and dupilumab response may represent a previously unrecognized biological mechanism.

*RNF135* (Ring finger protein 135) plays an important role in innate immune signaling by promoting RIG-I–mediated interferon responses during viral infection [35,36,37,38]. This immune regulatory function suggests that *RNF135* may influence inflammatory pathways, making its identification in our analysis particularly intriguing despite its absence from enriched GO terms. Given the central role of interferon and innate immune signaling in inflammatory diseases, our findings may point to a previously unrecognized mechanism contributing to variability in dupilumab treatment response.

Finally, exploratory external dataset comparison demonstrated partial overlap with previously identified differentially expressed genes, although directionality of expression was not fully concordant between datasets. Therefore, these findings should be interpreted as evidence that related immune-associated transcriptional programs involving overlapping genes may be captured across datasets, rather than as direct replication of individual gene-level effects. Importantly, RT-qPCR showed concordant expression direction for *BUB1B*, *AURKA*, and *RPS28* compared with the RNA-seq analysis, providing additional supportive evidence for these candidate genes despite the limited statistical power of the cohort. Although statistically significant expression differences were not observed after logistic regression adjustment, *BUB1B* demonstrated a suggestive association with treatment response and remained consistently downregulated in non-responders across analytical approaches.

Nevertheless, our study has several limitations. First, the relatively small sample size may reduce statistical power and limit the generalizability of the findings. In particular, the limited number of non-responders increases the possibility of false-positive findings and reduces statistical stability across transcriptomic and genomic analyses. The limited sample size also affects the stability of WGCNA-derived modules; therefore, the WGCNA results should be interpreted as exploratory co-expression patterns rather than definitive network structures. Second, Mendelian randomization analyses should be interpreted primarily as exploratory prioritization approaches, preventing formal sensitivity analyses such as MR-Egger, weighted median estimation, heterogeneity testing, or pleiotropy assessment. Third, blood-based biomarkers may not fully capture local cutaneous pathophysiological processes in AD, potentially limiting biological interpretation. Finally, RT-qPCR validation was also performed on a limited cohort, reducing the statistical power and robustness of validation analyses. Consequently, all identified candidate biomarkers should be interpreted cautiously until validated in larger independent cohorts.

In conclusion, this study applied a multi-stage integrative analytical framework combining transcriptomic profiling, functional enrichment, co-expression network analysis, and genomic variant analysis to identify potential biomarkers associated with dupilumab treatment response in patients with AD. Through systematic filtering of therapy- and inflammation-related signals, we identified a set of candidate genes and genomic variants that may reflect underlying biological mechanisms influencing therapeutic response. Several of the identified genes are functionally linked to pathways involved in immune regulation, cellular proliferation, and PI3K/AKT/mTOR signaling, supporting their potential relevance in AD pathophysiology. Although the present findings are exploratory and limited by sample size, convergent signals across multiple analytical approaches consistently highlighted overlapping candidate genes. Because nominal *p*-value thresholds were used in several downstream analyses, the possibility of false-positive findings cannot be excluded. Therefore, the identified genes and pathways should primarily be considered hypothesis-generating candidate signals requiring validation in larger independent cohorts rather than clinically validated predictive biomarkers. Overall, our findings provide additional insight into molecular mechanisms potentially underlying variability in dupilumab response and may support future development of personalized therapeutic strategies in AD.

## 4. Materials and Methods

### 4.1. Ethical Approval

For the purposes of the study, ethical approval was obtained from the Ethics Committee of the Republic of Slovenia (ethical approval number: KME 0120-433/2023/3). The study was conducted in accordance with the principles of the Declaration of Helsinki. All participants received information about the course of the study prior to voluntarily signing the informed consent form to participate in the research.

### 4.2. Enrolled Subjects

All enrolled patients were of Caucasian Central European ethnicity. The study included patients who were treated at the Department of Dermatovenerology at the University Medical Center Maribor and had been diagnosed with AD for at least 12 months. To be classified as having moderate-to-severe AD, dupilumab-naïve patients had to meet at least one of the following criteria: EASI ≥ 7.1, vIGA-AD ≥ 3, BSA ≥ 10%, or a pruritus score of ≥4 on the numeric VAS scale. Patients with immunodeficiency and those who were concomitantly treated with other systemic therapies for AD were excluded from the study. Additionally, among dupilumab-naïve patients, those who had received systemic corticosteroids, methotrexate, mycophenolate, or other immunosuppressive agents within the previous 4 weeks were excluded.

In addition, nine healthy controls were also included in the present study. Healthy controls had been previously recruited at the Center for Human Molecular Genetics and Pharmacogenomics, Faculty of Medicine, University of Maribor.

For an RNA-seq experiment, we enrolled eighteen patients with moderate-to-severe AD, who were naïve to dupilumab treatment. Peripheral venous blood samples were collected at baseline and 16–18 weeks after initiation of dupilumab therapy. Out of the eighteen patients, five were classified as non-responders, defined as not achieving EASI75. Transcriptomic profiles of peripheral blood from nine healthy controls were used to identify differentially expressed genes associated with inflammation and treatment.

The genotyping analysis included 34 patients with AD, comprising 18 patients from the initial cohort (13 responders and 5 non-responders) and 16 additional patients classified as long-term good responders to dupilumab, defined as maintaining a good response for 1–3 years. Demographic data and clinical parameters for patients are presented in Table 6 and Table 7, respectively.

### 4.3. DNA and RNA Isolation

DNA and RNA were extracted from peripheral blood mononuclear cells using the Monarch^®^ Spin gDNA Extraction Kit (New England Biolabs, Ipswich, MA, USA) and the innuPREP Micro RNA Kit (iST Innuscreen GmbH, Berlin, Germany), respectively. DNA and RNA with sufficient concentration and quality were further included in downstream analyses.

### 4.4. RNA Sequencing and Differential Gene Expression Analysis

RNA samples were sequenced at BGI facilities using BGI Optimal Dual-mode mRNA Library Prep Kit PE150 on the DNBSEQ G400 (BGI, Shenzhen, China). Raw data containing adapter sequences or low-quality reads were removed using the SOAPnuke tool by BGI [39].

A three-stage approach was employed for differential expression analysis, similar to that used in a previously published article by our group [40]. In stage one, differentially expressed genes influenced by dupilumab therapy were identified. These genes were excluded prior to the second stage. Stage two consisted of two substages aimed at identifying differentially expressed genes associated with inflammation. The union of unique differentially expressed genes identified in both substages was considered to represent inflammation-related genes and was subsequently removed from the analysis prior to the third stage. In the final stage, differentially expressed genes potentially associated with response to dupilumab were determined.

Blood transcriptome analysis for each stage was performed using a custom pipeline developed by the Center for Human Molecular Genetics and Pharmacogenomics in the environment R 4.5.2 (R Core Team, Vienna, Austria), employing various packages as described in previously published studies [41]. In stage one, genes with an adjusted *p*-value < 0.05 were considered differentially expressed. In all subsequent stages, a *p*-value < 0.05 was used as the significance threshold. These downstream analyses were intended as exploratory prioritization steps within the integrative framework rather than definitive transcriptome-wide inference. Therefore, nominal significance thresholds were used to avoid excessive signal loss in the context of a limited sample size.

### 4.5. Gene Ontology Analysis

Gene ontology (GO) analysis was performed in R 4.5.2 using the *clusterProfiler 4.16.0* package [42,43]. Importantly, GO was conducted separately for significant up- and downregulated differentially expressed genes. Genes with log_2_FC > 0 represented upregulated genes, while those with log_2_FC < 0 indicated downregulated ones. Enrichment analysis was carried out for molecular function and biological processes, with significance thresholds set at *p*-value < 0.05 and *q*-value < 0.1. Genes listed at enriched GO terms were extracted and subsequently excluded from the set of significant differentially expressed genes to identify non-GO-enriched genes.

### 4.6. Gene Scores, Logistic Regression, and Weighted Gene Co-Expression Network Analysis

After GO analysis, gene scores for each GO term and for non-GO-enriched genes were calculated for each patient. To avoid collinearity in logistic regression, bias-reduced logistic regression was performed in R 4.5.2 using the *brglm2 1.0.1* package [44]. The logistic regression model was adjusted for age and sex. Significant gene scores for GO terms and non-GO-enriched genes were obtained from the logistic regression results. Genes in significant GO terms were further used for targeted genomic profiling. In addition, significant gene scores (*p*-value < 0.05) for non-GO-enriched genes were further analyzed using weighted gene co-expression network analysis (WGCNA) in R 4.5.2 using the *WGCNA 1.74* package [45]. Identified hub genes were defined with module membership (kME) greater than 0.9.

### 4.7. Genomic Profiling and Association Analysis

For 34 DNA samples, genotyping was performed. Genotyping was conducted with Illumina Infinium Global Screening Array V4.0 (Illumina Inc., San Diego, CA, USA) by the Institute of Clinical Molecular Biology in Kiel, Germany. Extensive quality control was carried out as previously described [46]. Genotype imputation was performed using a TOPMed imputation server and a TOPMed r3 panel with the Minimac4 imputation algorithm, an Rsq filter of >0.3, and Eagle 2.4 phasing [47,48,49]. Association analysis was performed between non-responders and responders using Wald logistic regression implemented in PLINK 2.0 software, and using genotypic dosages [50]. An association analysis was conducted using a regression model adjusted for age and sex. Targeted genomic profiling and integration with genomic data were performed by extracting variants located within ±100 kb of previously identified and enriched differentially expressed genes from RNA-seq and GO analyses, respectively. Variants were considered statistically significant at *p*-value < 0.05, prior to identifying independent variants through LD pruning using the SNPclip tool from LDlink software 6.0.0, with a threshold R^2^ of 0.5 [51]. Variants residing in selected genomic regions are presented in regional Manhattan plots constructed using LocusZoom [52]. Tissue expression quantitative trait loci (eQTLs) were estimated using the GTEx Portal Database (https://www.gtexportal.org/home/, accessed on 5 March 2026) or the eQTL Catalogue (https://www.ebi.ac.uk/eqtl/, accessed on 5 March 2026). When the gene or SNP of interest was not available in the GTEx portal or the eQTL Catalogue, proxy SNPs (D’ > 0.7) were identified using the LDproxy tool from the LDlink software 6.0.0 and examined for expression in blood or blood cells [51].

Additionally, two-sample Mendelian randomization (MR) analysis was conducted using the inverse-variance weighted (IVW) random-effects model implemented in the *MendelianRandomization 0.10.0* R package [53]. MR analyses were performed for genes for which eQTL data in blood or blood cells were available. The corresponding SNPs, or their highest linkage disequilibrium (LD) proxies when eQTLs were reported, were used as instrumental variables. eQTL effect sizes with their corresponding standard errors were used as variant–exposure associations, while beta coefficients and their respective standard errors from association analyses were used as variant–outcome associations. The causal effect of gene expression on dupilumab treatment response was considered statistically significant at *p*-value < 0.05. Because only one blood-related eQTL instrument was available for each gene, causal inference from MR analyses remains limited and should be interpreted cautiously.

### 4.8. Exploratory External Dataset Comparison

Finally, an independent dataset was used for exploratory external comparison of the prioritized transcriptional signals [54]. The dataset was downloaded from the GEO database, specifically GSE208405 (https://www.ncbi.nlm.nih.gov/geo/query/acc.cgi?acc=GSE208405, accessed on 4 May 2026). Before the differential gene expression analysis, the samples were filtered, and only those from patients with AD whose blood samples had been taken prior to dupilumab therapy were selected. Together, 46 samples were included in the downstream analysis.

Differential gene expression analysis was conducted as described in the aforementioned paragraph. Absolute immune deconvolution estimates derived from publicly available RNA-seq data were used to identify eosinophil-enriched (“eosinophil-hot”) samples. Samples with detectable eosinophil infiltration were operationally classified as non-responders (N = 6) based on observations reported in the original study associated with the dataset [54].

### 4.9. RT-qPCR Assessment of Candidate Genes

Nucleotide sequences of the exons of the target genes were obtained from the NCBI Gene database (https://www.ncbi.nlm.nih.gov/gene/, accessed on 4 May 2026). Isoform non-specific primers were hand-picked and designed using Primer-BLAST (https://www.ncbi.nlm.nih.gov/tools/primer-blast/, accessed on 4 May 2026) [55] and Primer3 [56]. Primers for reference genes *ACTB* and *B2M* were obtained from a previous study [57]. Primer sequences are summarized in Table 8. All primers were synthesized by Sigma-Aldrich (Merck KGaA, Darmstadt, Germany). mRNA was obtained from 18 (5 non-responders and 13 responders) enrolled individuals as described in the aforementioned paragraph. A concentration of 50 ng/µL of mRNA was transcribed into cDNA using the High-Capacity cDNA Reverse Transcription Kit (Applied Biosystems^TM^, Thermo Fisher Scientific, Waltham, MA, USA). Next, 2 µL of 10-fold diluted cDNA was used as a template. RT-qPCR gene expression assay was carried out using Lightcycler 480^®^ SYBR Green I Master Mix and Lightcycler 480^®^ real-time thermocycler (Roche, Basel, Switzerland) according to the manufacturer’s instructions. Melting curves for each sample were analyzed after each run in order to confirm the specificity of amplification. Raw Ct values were obtained from two run-independent technical replicates for each sample. Normalization of raw data was performed using geometric averaging of reference genes, and relative expression was calculated using the 2^−ΔΔCt^ method [58]. Stability of reference genes was statistically assessed using the relative 2^−ΔCt’^calculation. In addition, logistic regression was conducted using IBM SPSS Statistics (Version 31, IBM Corp., Armonk, NY, USA). Analysis was adjusted for sex and age. Statistical significance was defined as a *p*-value < 0.05.

## Figures and Tables

**Table 1:** WGCNA results.

Module	Module Color	Number of Genes in the Module	*p*-Value for the Module
0	gray	14	0.0584
1	turquoise	109	0.0734
2	blue	104	0.0519
3	brown	53	0.0657
4	yellow	46	0.0551

**Table 2:** Identified variants.

Chr	Base Pair (GRCh38)	dbSNP ID	A1	Gene	*p*-Value
19	17,831,128	rs3212777	G	*RPL18A*	0.0339
19	8,315,265	rs8110376	C	*RPS28*	0.0454
11	65,157,627	rs75018393	T	*FAU*	0.0432
10	27,250,013	rs590142	C	*MASTL*	0.0409
20	56,295,713	rs6069696	A	*AURKA*	0.0322
8	119,695,360	rs9297602	G	*TAF2*	0.0265
15	40,157,612	rs55940969	T	*BUB1B*	0.0454
2	175,160,299	rs2788522	T	*ATF2*	0.0219
2	175,037,495	rs12613111	C	*ATF2*	0.0416
17	30,925,135	rs11868576	T	*RNF135*	0.0421

Chr: chromosome; A1: effect allele.

**Table 3:** eQTL of selected variants.

dbSNP ID (Gene)	Proxy SNP	D’	Tissue	*p*-Value
rs3212777 (*RPL18A*)	rs111922843	0.7083	blood ^†^	6.60 × 10^−13^
rs8110376 (*RPS28*)	rs2913944	1	whole blood ^‖^	1.70 × 10^−8^
rs75018393 (*FAU*)	rs2957879	1	whole blood ^‖^	6.00 × 10^−5^
rs590142 (*MASTL*)	/	/	whole blood ^‖^	5.20 × 10^−14^
rs6069696 (*AURKA*)	rs2298016	0.9201	macrophage: IFNg 18 h ^†^	6.88 × 10^−10^
rs9297602 (*TAF2*)	rs16892938	1	whole blood ^‖^	6.40 × 10^−6^
rs55940969 (*BUB1B*)	rs62020025	1	whole blood ^‖^	5.00 × 10^−5^
rs11868576 (*RNF135*)	rs6505219	0.9047	whole blood ^‖^	8.30 × 10^−5^

^‖^ Data obtained from the GTEx portal; ^†^ Data obtained from the eQTL Catalogue.

**Table 4:** Results of the independent dataset.

Gene	log_2_FC	CI ^‖^	*p*-Value
*RPL18A*	−0.4809	−1.0318 to 0.0700	0.0855
*RPS28*	−0.8069	−1.5704 to −0.0433	0.0388
*FAU*	−0.4038	−0.8955 to 0.0879	0.1051
*MASTL*	0.1165	−0.1939 to 0.4270	0.4538
*AURKA*	0.2827	−0.1012 to 0.6666	0.1451
*TAF2*	0.3597	−0.0347 to 0.7542	0.0729
*BUB1B*	0.4093	−0.1405 to 0.9591	0.1408
*RNF135*	−0.0904	−0.3090 to 0.1281	0.4091

^‖^ 95% confidence interval.

**Table 5:** Relative gene expression between non-responders and responders.

Gene	2^−ΔΔCt^	Minimal Error	Maximal Error	*p*-Value ^‖^
*RPL18A*	1.149	0.963	1.372	0.139
*RPS28*	1.526	0.778	2.995	0.299
*FAU*	1.050	0.885	1.245	0.562
*MASTL*	0.967	0.690	1.356	0.406
*AURKA*	0.655	0.529	0.812	0.386
*TAF2*	1.128	0.630	2.020	0.803
*BUB1B*	0.724	0.503	1.042	0.099
*RNF135*	1.179	0.950	1.463	0.326

^‖^ *p*-value from logistic regression analysis.

**Table 6:** Demographic data for patients included in the study.

	Naïve Patients (N = 18)	Long-Term Good Responders (N = 16)	Healthy Controls (N = 9)
Mean age at time of sampling (SD)	27.44 (9.20)	31.88 (13.92)	43.11 (6.58)
Sex, N (%)			
Female	6 (33.3%)	7 (43.8%)	4 (44.4%)
Male	12 (66.7%)	9 (56.3%)	5 (55.6%)
Treatment outcome, N (%)			
Response	13 (72.2%)		
Non-response	5 (27.8%)		

SD: Standard deviation.

**Table 7:** Clinical parameters for dupilumab-naïve patients.

	Naïve Patients (N = 18)		
	Baseline Parameters	Follow-Up Parameters	*p*-Value *
BSA [%]			
Mean (SD)	30.39 (25.20)	3.28 (4.06)	<0.001
Median	20	1.50	
Minimum	5	0	
Maximum	90	12	
vIGA-AD			
Mean (SD)	2.81 (0.54)	0.69 (0.70)	<0.001
Median	3	1	
Minimum	2	0	
Maximum	4	2	
VAS pruritus			
Mean (SD)	7.44 (1.88)	2.56 (2.20)	<0.001
Median	7.50	2.50	
Minimum	3	0	
Maximum	10	7	
VAS sleep disturbance			
Mean (SD)	5.67 (3.42)	0.72 (1.67)	0.002
Median	8	0	
Minimum	0	0	
Maximum	10	6	
EASI			
Mean (SD)	16.96 (9.09)	1.60 (2.13)	<0.001
Median	15.85	0.55	
Minimum	5.10	0	
Maximum	39.50	5.70	

* Non-parametric Wilcoxon signed-rank test; BSA: body mass index; vIGA-AD: validated investigator global assessment scale for atopic dermatitis; VAS: visual analog scale; EASI: eczema area and severity index.

**Table 8:** Primer sequences of target and reference genes.

Gene	Forward Primer (5′–3′)	Reverse Primer (5′–3′)
*TAF2*	ACTTCTTCCGAGTTACAGGCA	TGGTGGGTTCTTAGTCAACATG
*MASTL*	TGCATCCAATAACTCAGAACCA	AATTCGCCCATCATCAACGG
*FAU*	CATTGCCCCGGAAGATCAAG	TAGCCCGACCTGTCTTCTTC
*BUB1B*	GATCATGTCCACGCTTCAGG	ACTCTCCTTCCCACCTTGAG
*RNF135*	CAACGAACTGAGCATCCTGG	ATGGGCATGAGGAGGAAGAC
*RPS28*	CGTGTGCAGCCTATCAAGC	CTCGCTCTGACTCCAAAAGG
*RPL18A*	GGAGATTGTCTACTGTGGGC	GCGATCTCCTCCACCTTCAT
*AURKA*	AATTCTTCCCAGCGCATTCC	AAGTCTTCCAAAGCCCACTG
*B2M* ^†^	TTCTGGCCTGGAGGCTATC	TCAGGAAATTTGACTTTCCATTC
*ACTB* ^†^	CATCGAGCACGGCATCGTCA	TAGCACAGCCTGGATAGCAAC

^†^ Reference genes.

## Data Availability

The original data presented in the study are openly available in the Gene Expression Omnibus (GEO) repository at GSE326320.

## Supplementary Materials

The following supporting information can be downloaded at: https://www.mdpi.com/article/10.3390/ijms27115147/s1.

## Abbreviations

The following abbreviations are used in this manuscript:

AD	Atopic dermatitis
AUC	Area under the curve
BSA	Body surface area
EASI	Eczema Area and Severity Index
eQTL	Tissue expression quantitative trait loci
GO	Gene ontology
MR	Mendelian randomization
ROC	Receiver operating characteristic
VAS	Visual analog scale
vIGA-AD	Validated Investigator Global Assessment for Atopic Dermatitis
WGCNA	Weighted Gene Co-Expression Network Analysis
