# Supplementary material for: Integrative Transcriptomic, Network, and Genomic Analysis of Peripheral Blood Mononuclear Cells Identifies Candidate Genes Associated with Dupilumab Clinical Response in Atopic Dermatitis Patients
Source: Int J Mol Sci. 2026 Jun 5;27(11):5147. doi: 10.3390/ijms27115147 (PMC13258615; doi:10.3390/ijms27115147)
Supplement: Supplementary file 1 [file ijms-27-05147-s001.zip › Figures_S3_S11.pdf]

Figure S3: Regional Manhattan plot for *ATF2*.

Figure S4: Regional Manhattan plot for *AURKA*.

Figure S5: Regional Manhattan plot for *BUB1B*.

Figure S6: Regional Manhattan plot for *FAU*.

Figure S7: Regional Manhattan plot for *MASTL*.

Figure S8: Regional Manhattan plot for *RNF135*.

Figure S9: Regional Manhattan plot for *RPL18A*.

Figure S10: Regional Manhattan plot for *RPS28*.

Figure S11: Regional Manhattan plot for *TAF2*.

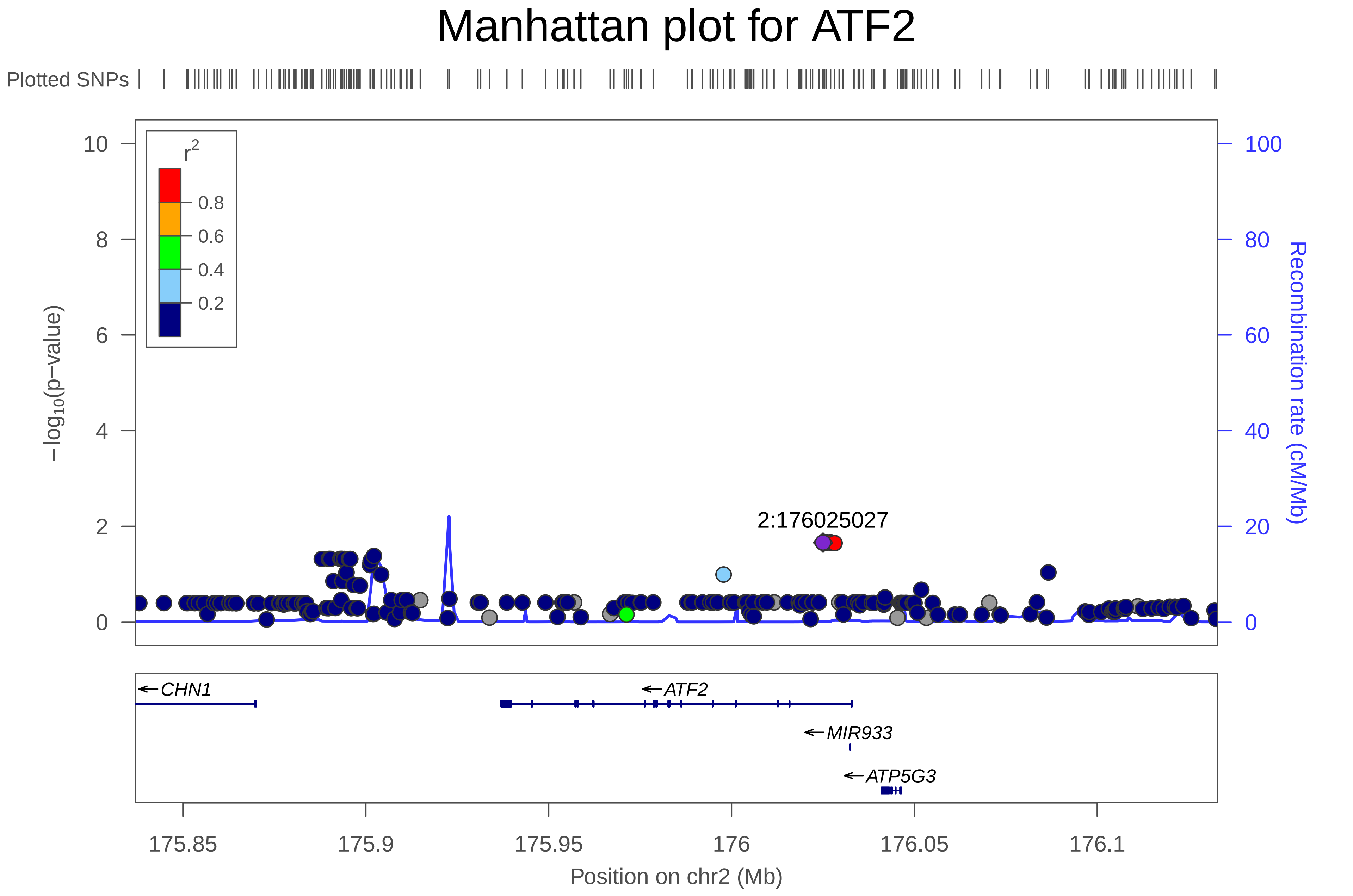

Manhattan plot for ATF2
Plotted SNPs
r2
0.8
0.6
0.4
0.2
10
8
6
4
2
0
−log10(p−value)
100
80
60
40
20
0
Recombination rate (cM/Mb)
2:176025027
CHN1
ATF2
MIR933
ATP5G3
175.85
175.9
175.95
176
176.05
176.1
Position on chr2 (Mb)

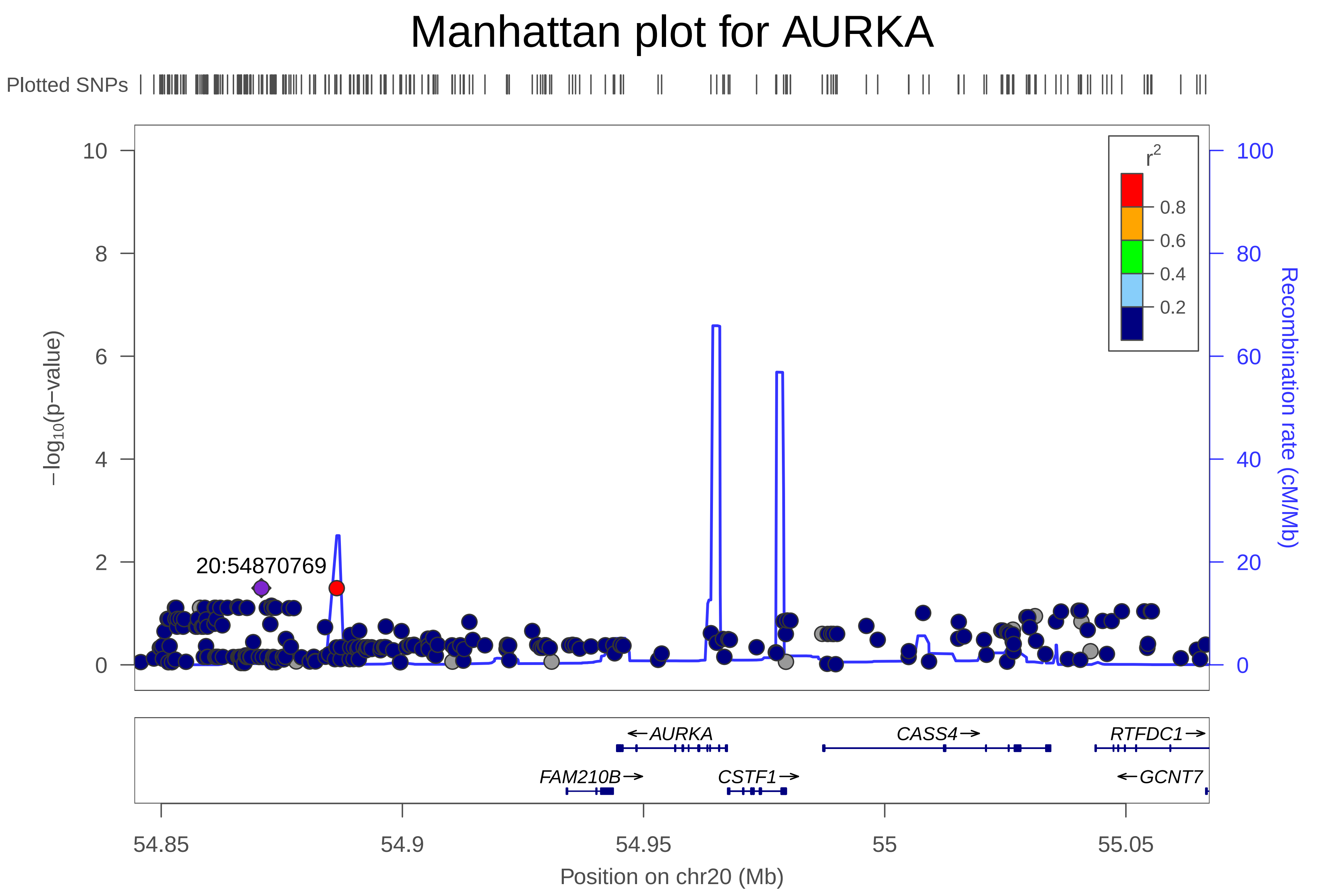

Manhattan plot for AURKA
Plotted SNPs
−log10(p−value)
Recombination rate (cM/Mb)
r2
0.8
0.6
0.4
0.2
20:54870769
AURKA
CASS4
RTFDC1
FAM210B
CSTF1
GCNT7
54.85
54.9
54.95
55
55.05
Position on chr20 (Mb)

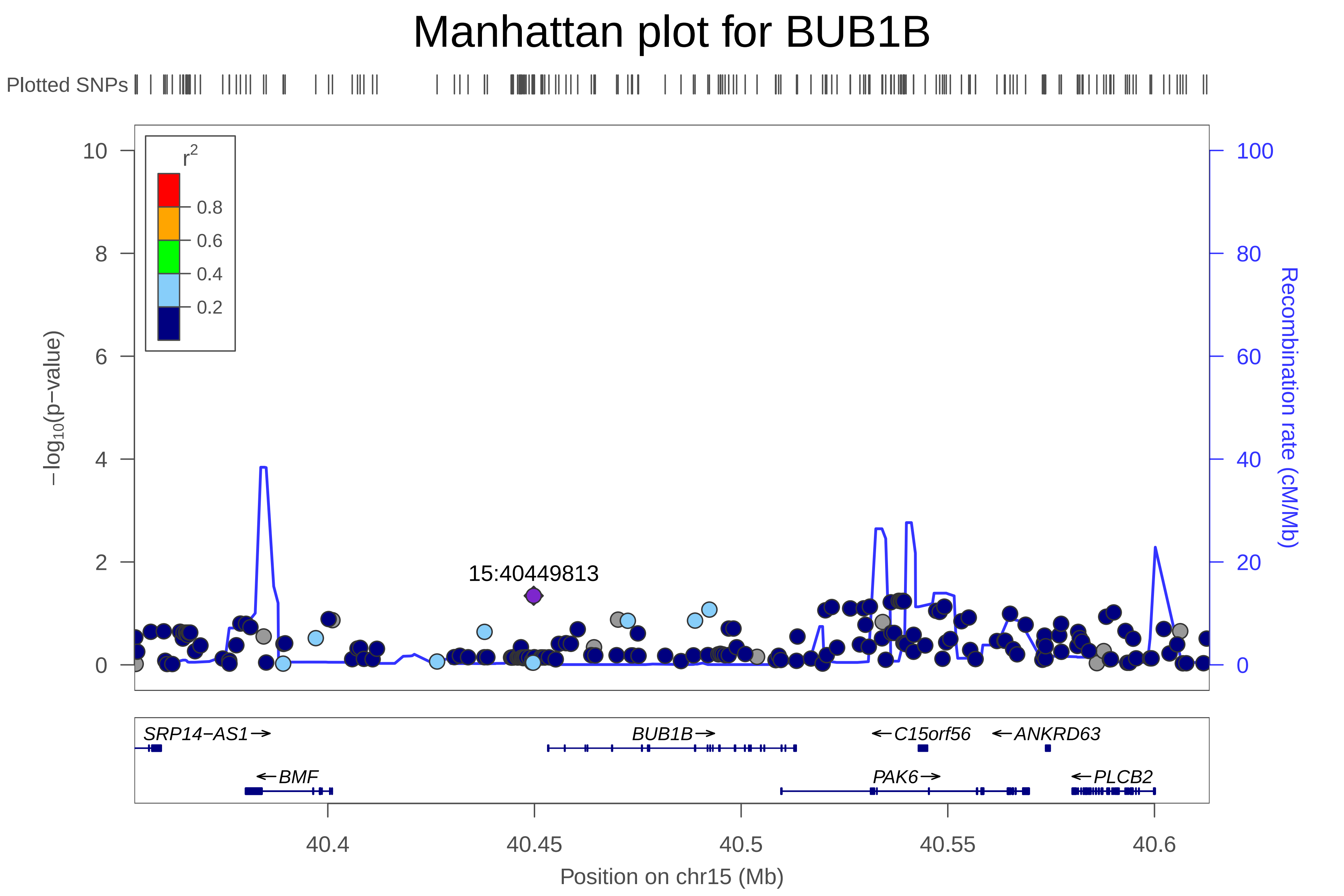

Manhattan plot for BUB1B
Plotted SNPs
r2
0.8
0.6
0.4
0.2
15:40449813
−log10(p−value)
Recombination rate (cM/Mb)
10
8
6
4
2
0
100
80
60
40
20
0
SRP14−AS1→
←BMF
BUB1B→
←C15orf56
PAK6→
←ANKRD63
←PLCB2
40.4
40.45
40.5
40.55
40.6
Position on chr15 (Mb)

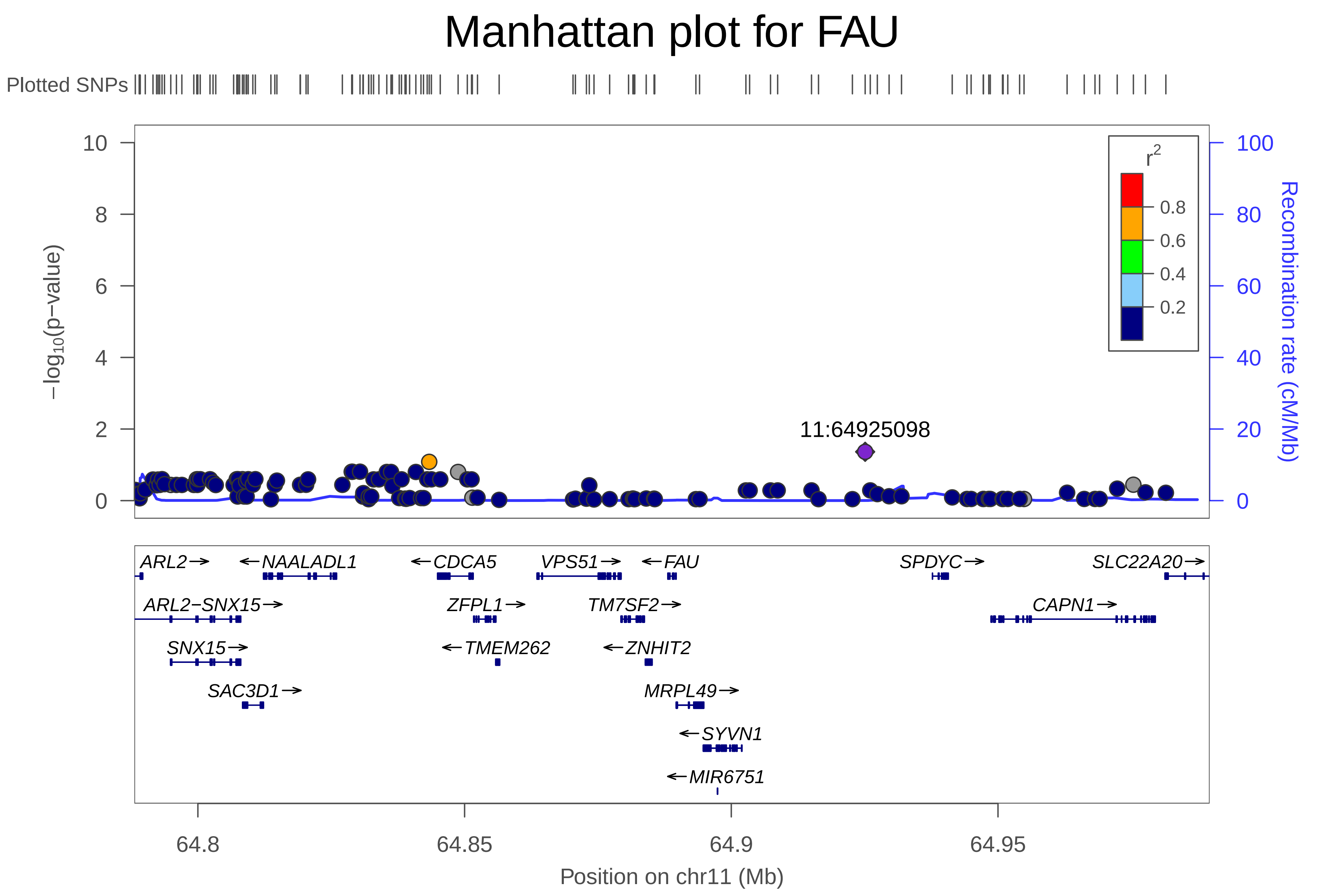

Manhattan plot for FAU
Plotted SNPs
$-\log_{10}$(p−value)
10
8
6
4
2
0
11:64925098
r^2
0.8
0.6
0.4
0.2
100
80
60
40
20
0
Recombination rate (cM/Mb)
ARL2→
←NAALADL1
←CDCA5
VPS51→
←FAU
SPDYC→
SLC22A20→
ARL2−SNX15→
ZFPL1→
TM7SF2→
CAPN1→
SNX15→
←TMEM262
←ZNHIT2
SAC3D1→
MRPL49→
←SYVN1
←MIR6751
64.8
64.85
64.9
64.95
Position on chr11 (Mb)

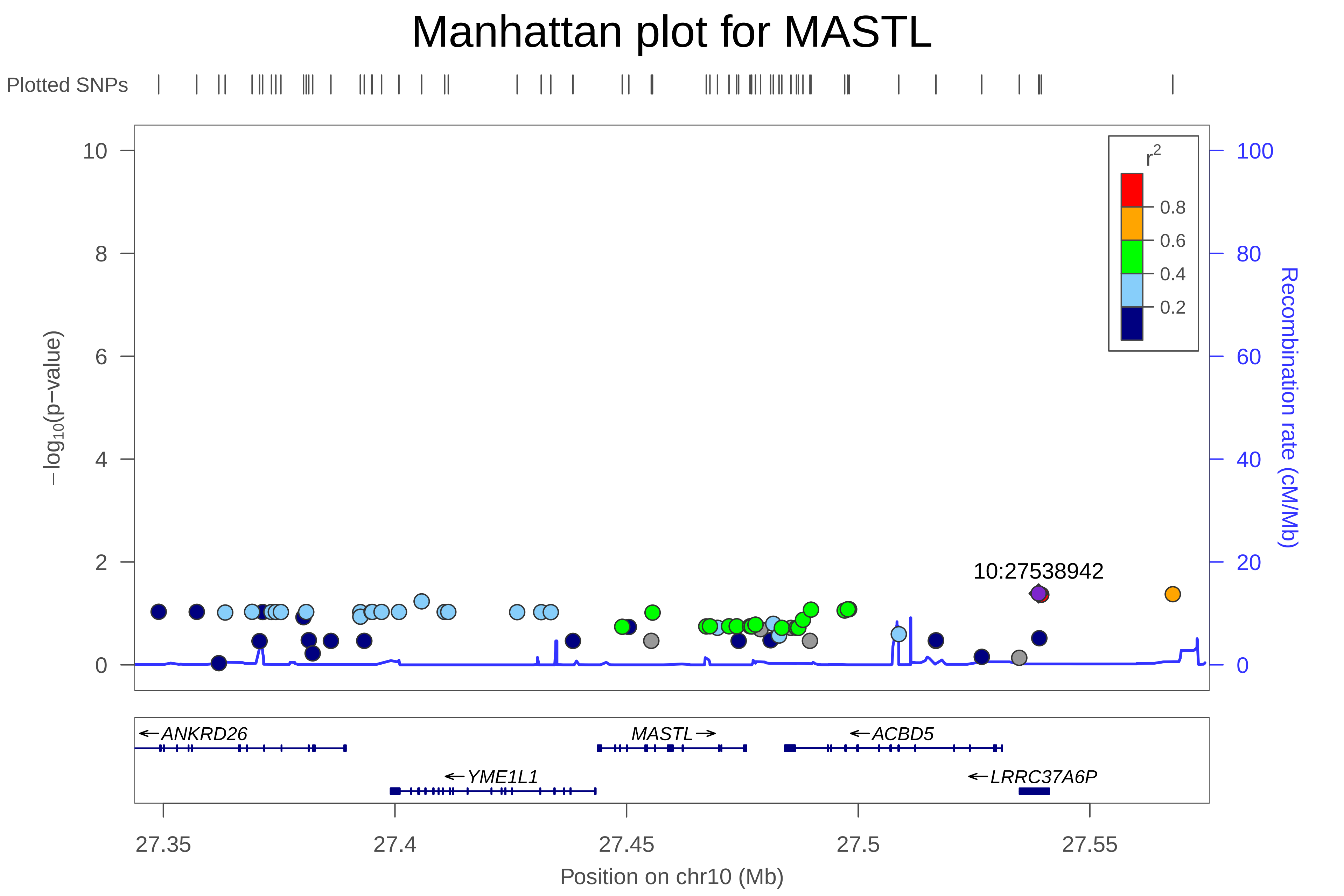

Manhattan plot for MASTL
Plotted SNPs
−log10(p−value)
Recombination rate (cM/Mb)
r2
0.8
0.6
0.4
0.2
10:27538942
ANKRD26
MASTL
ACBD5
YME1L1
LRRC37A6P
27.35
27.4
27.45
27.5
27.55
Position on chr10 (Mb)

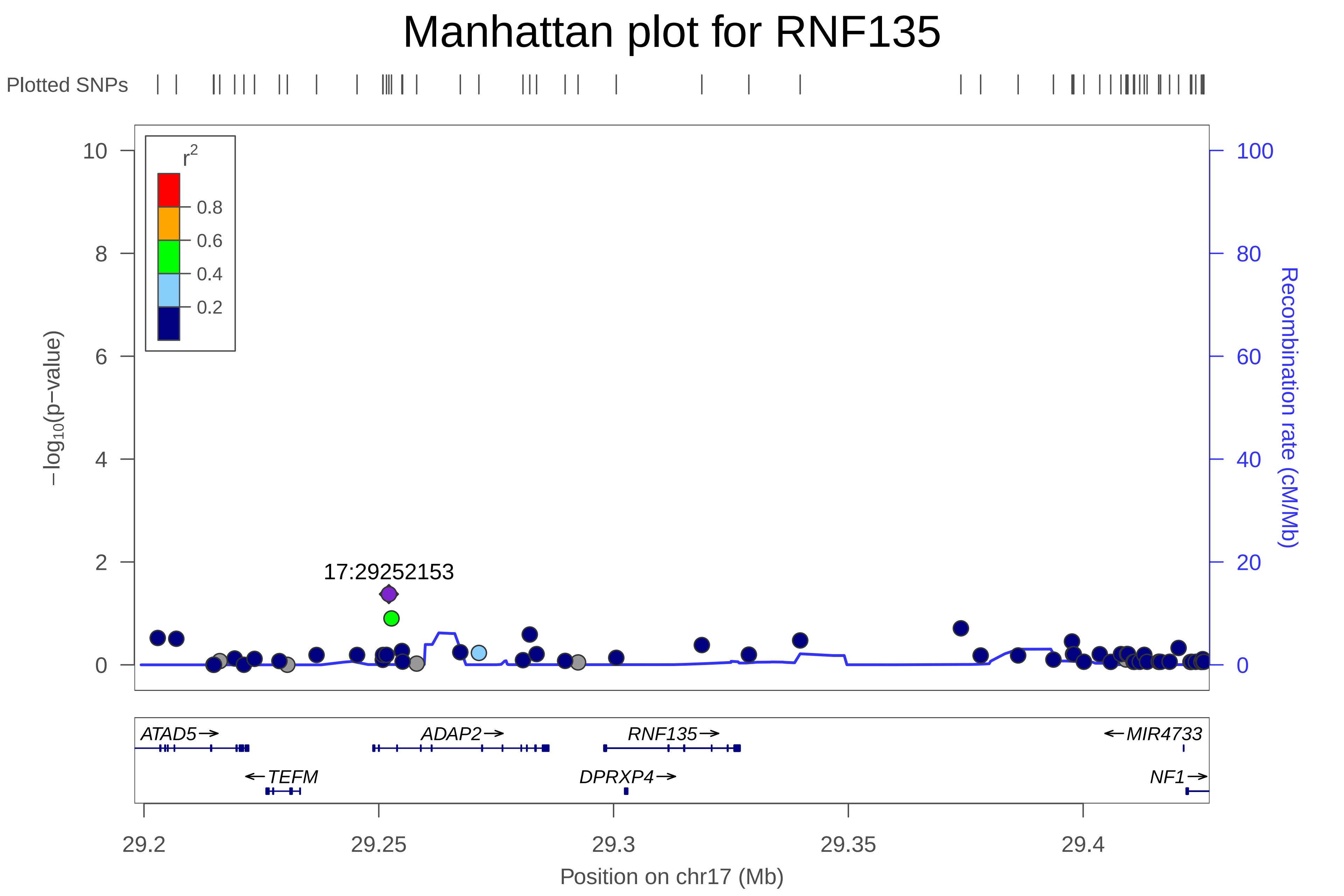

Manhattan plot for RNF135
Plotted SNPs
r2
0.8
0.6
0.4
0.2
10
8
6
4
2
0
−log10(p−value)
100
80
60
40
20
0
Recombination rate (cM/Mb)
17:29252153
ATAD5
TEFM
ADAP2
RNF135
DPRXP4
MIR4733
NF1
29.2
29.25
29.3
29.35
29.4
Position on chr17 (Mb)

Manhattan plot for RPL18A

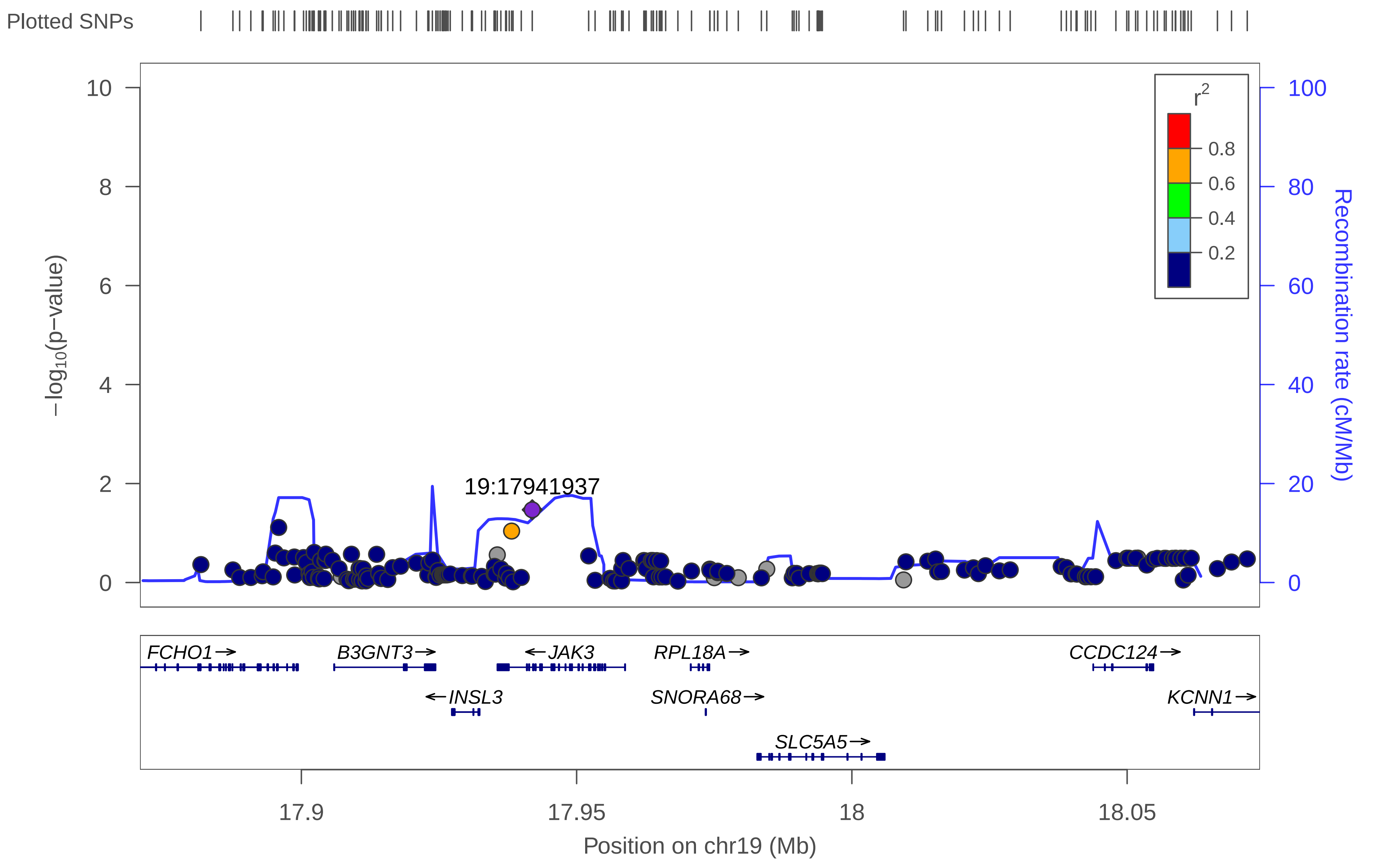

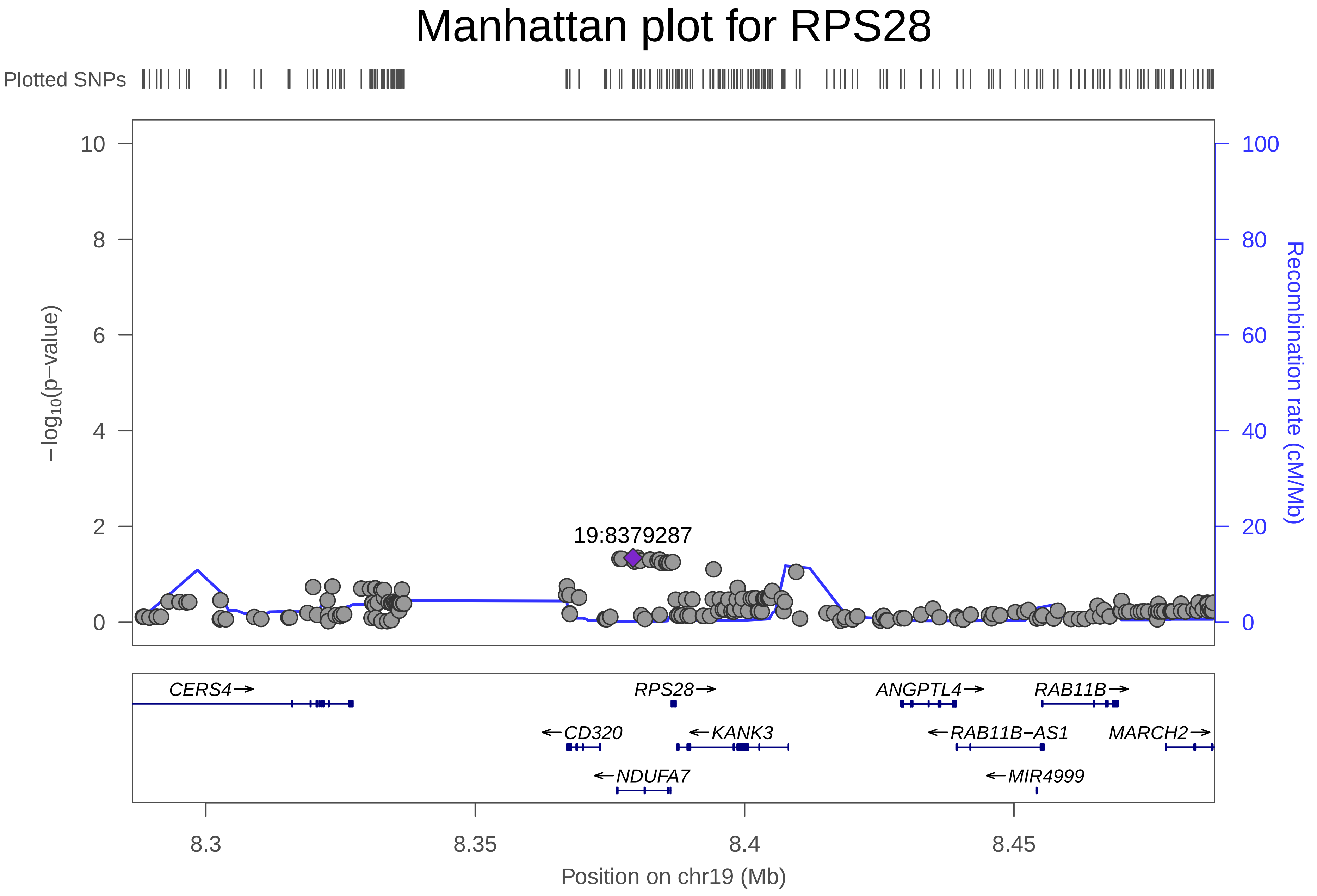

Manhattan plot for RPS28
Plotted SNPs
−log10(p−value)
Recombination rate (cM/Mb)
19:8379287
CERS4→
RPS28→
ANGPTL4→
RAB11B→
←CD320
←KANK3
←RAB11B−AS1
MARCH2→
←NDUFA7
←MIR4999
0
2
4
6
8
10
0
20
40
60
80
100
8.3
8.35
8.4
8.45
Position on chr19 (Mb)

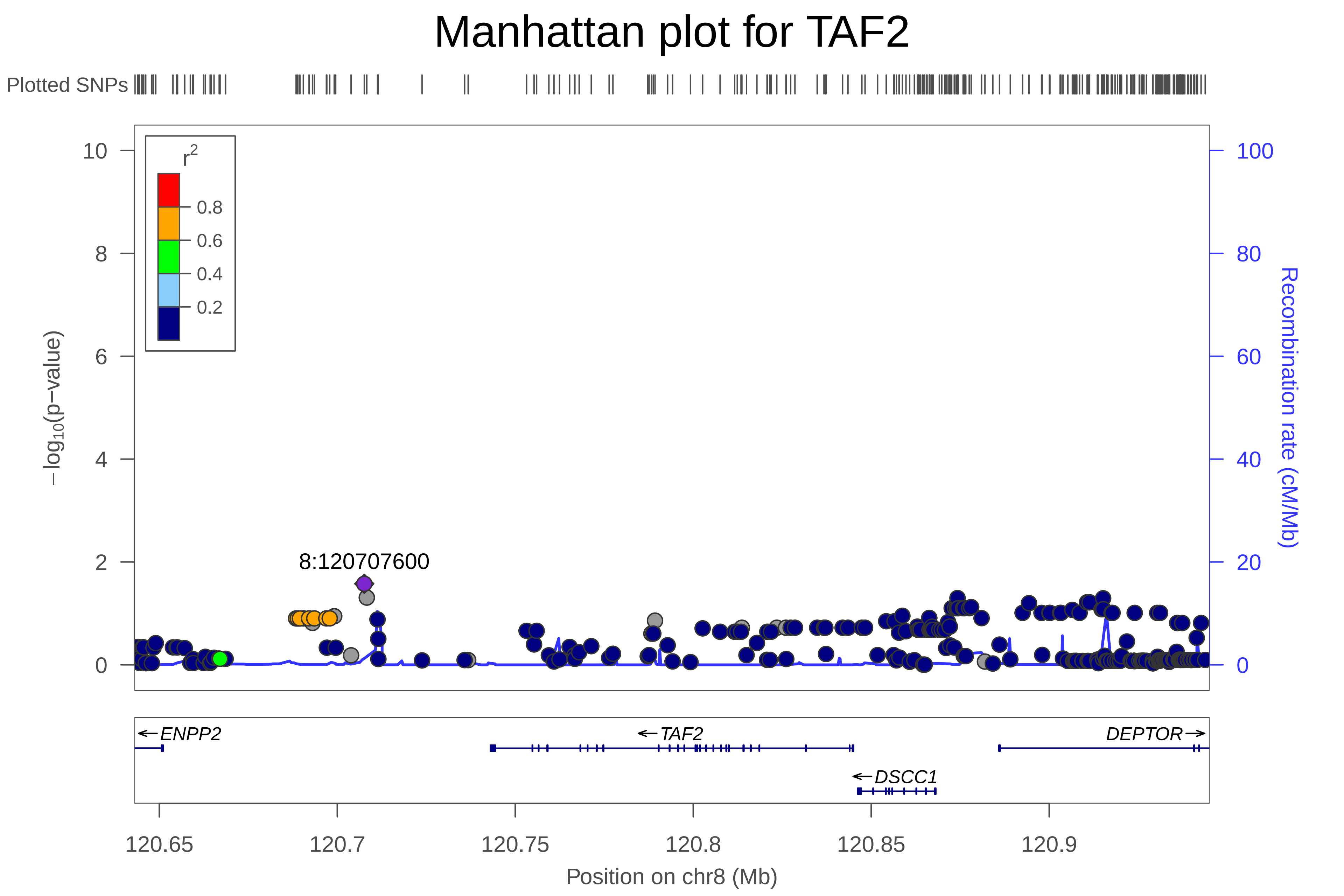
Manhattan plot for TAF2
Plotted SNPs
r2
0.8
0.6
0.4
0.2
8:120707600
−log10(p−value)
Recombination rate (cM/Mb)
ENPP2
TAF2
DEPTOR
DSCC1
120.65
120.7
120.75
120.8
120.85
120.9
Position on chr8 (Mb)
