# Supplementary figures and images for: Integrative Transcriptomic, Network, and Genomic Analysis of Peripheral Blood Mononuclear Cells Identifies Candidate Genes Associated with Dupilumab Clinical Response in Atopic Dermatitis Patients
Source: Int J Mol Sci. 2026 Jun 5;27(11):5147. doi: 10.3390/ijms27115147 (PMC13258615; doi:10.3390/ijms27115147)

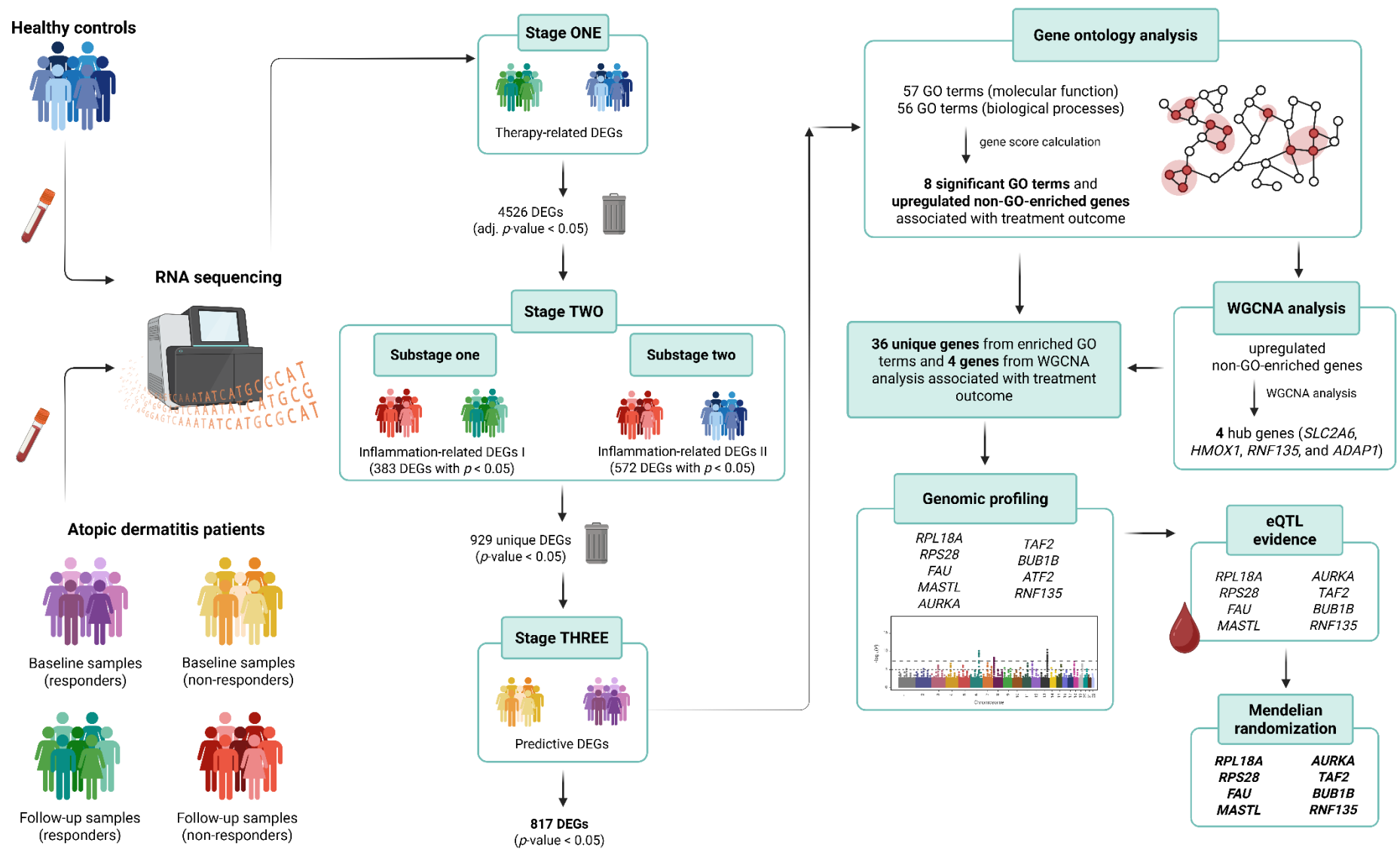

**Figure S1:** Detailed study design and workflow of the analysis (Created in BioRender).

Supplement: Supplementary file 1 [file ijms-27-05147-s001.zip › Figure_S1.pdf]

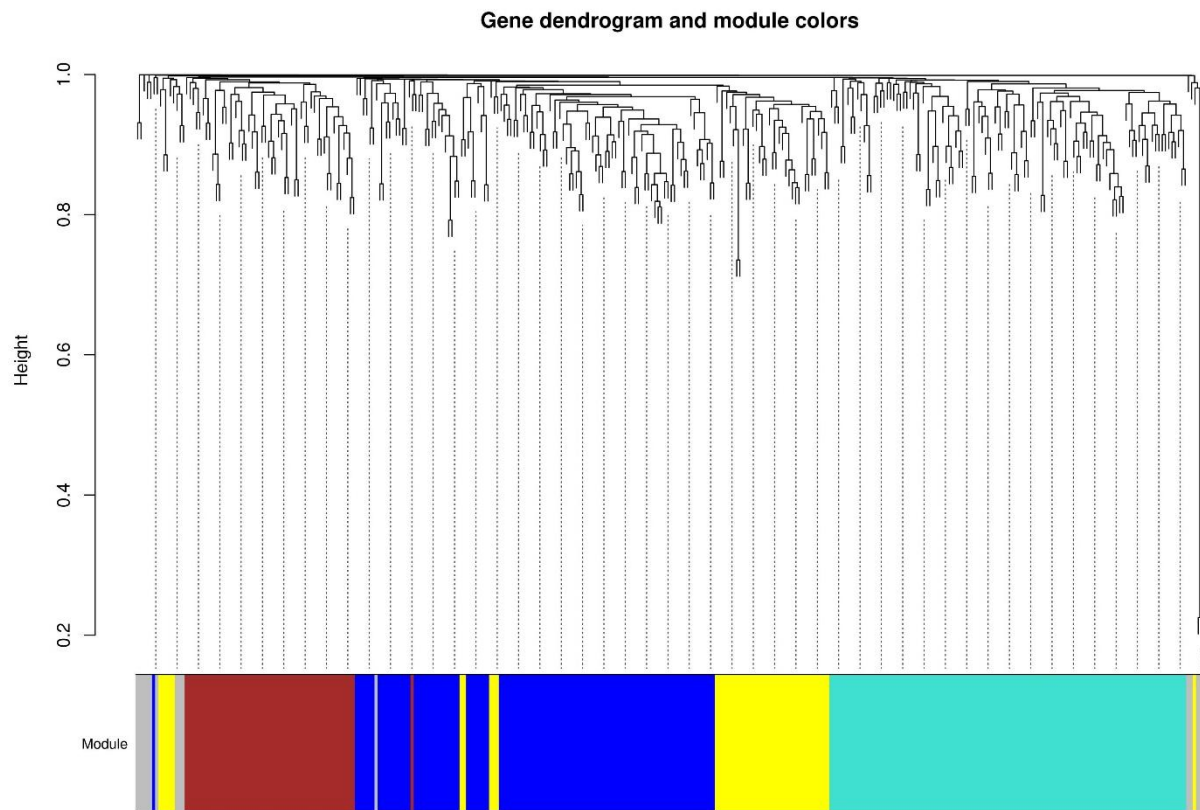

**Figure S2:** Gene dendrogram and module colors.

Supplement: Supplementary file 1 [file ijms-27-05147-s001.zip › Figure_S2.pdf]
